# The EPA Ecosystem Services Tool Selection Portal

**DOI:** 10.3390/su16051739

**Published:** 2024-02-20

**Authors:** Matthew C. Harwell, Leah M. Sharpe, Kaitlyn Hines, Cody Schumacher, Stephanie Kim, Gina Ferreira, Tammy A. Newcomer-Johnson

**Affiliations:** 1 Pacific Ecological Systems Division, US Environmental Protection Agency, Newport, OR 97365, USA; 2 Gulf Ecosystem Measurement and Modeling Division, US Environmental Protection Agency, Gulf Breeze, FL 32561, USA; 3 Contractor to US Environmental Protection Agency, Cincinnati, OH 45268, USA; 4 Region 2 Superfund and Emergency Management Division, US Environmental Protection Agency, New York, NY 10007, USA; 5 Watershed and Ecosystem Characterization Division, US Environmental Protection Agency, Cincinnati, OH 45268, USA

**Keywords:** ecosystem services, decision support, ecological risk assessment, contaminated site cleanup, decision-tree approach, user-centric design

## Abstract

The dynamics of an environmental decision-making context can be complicated. The use of decision support tools can help better facilitate restoring and maintaining ecosystems that provide environmental benefits (ecosystem services) to people. Although an ecosystem services assessment tool is designed for specific purposes, having access to a comprehensive suite of tools offers the user additional insight and resources to help in decision making. A range of approaches exist to connect ecosystem services to a given decision context ranging from less to more complex: using the best professional judgment; applying examples from other efforts; testing individual tool applications; and using a systematic, decision-tree approach to navigate among relevant tools and frameworks. The U.S. Environmental Protection Agency developed a decision-tree approach for a user to navigate the question of how to choose among a suite of ecosystem services assessment tools for three decision contexts: (1) ecological risk assessments; (2) cleanup of contaminated sites; (3) and generic structured decision-making processes. This tool selection navigator was developed with/for the intended user, including developing crosswalks between tool functionality and the user’s language for what they require in a tool. To navigate the tool, the user first chooses one of three decision contexts. Second, the user selects among the different phases of the decision process. Third, the user selects among a few ecosystem-services related tasks relevant to the decision context chosen to identify potential tools. The tool uses simple language to navigate the decision pathways and provides the user with a suite of potential ES resources and tools for their given decision context.

## 1. Introduction

Ecosystem goods and services (abbreviated here as ES) contribute to our well-being and the well-being of our communities in ways that often are not recognized until they are negatively impacted. U.S. Environmental Protection Agency (EPA) researchers have spent the last two decades advancing the science of ES, including the development of a number of different ES assessment decision-support tools (see below) to help project teams examine and incorporate ES benefits into their environmental planning and decision processes. Integrating ES assessment tools into the decision-making process where appropriate can increase the visibility of these benefits, strengthening community and environmental decision-making efforts.

It can be challenging for risk assessors, contaminated site cleanup practitioners, or others interested in environmental decision making to figure out the best ES assessment tool(s) to utilize. Many approaches exist for connecting ES to a given environmental decision-making context/process. In order of less to more complex, they range from: using best professional judgment, e.g., [1]; finding examples from other efforts to apply; testing individual tool applications; and using a systematic, decision-tree approach, e.g., [2,3] to navigate among potentially relevant ES tools and frameworks applicable to that decision-making process. While examples exist for the first two approaches, the third approach to navigate among ES tools does not exist in the literature. To address these challenges, the decision-tree approach was developed into a tool selection portal for a range of decision contexts, using a translational science approach focused on the needs of the potential users. The EPA Ecosystem Services (ES) Tool Selection Portal (https://www.epa.gov/eco-research/ecosystem-services-tool-selection-portal, accessed on 21 January 2024) (abbreviated here as the Portal) was co-developed with end-users to help guide environmental decision makers, ES scientists, and others in choosing among the most relevant suite of ES assessment tools, frameworks, and methodologies for application in a range of decision-making contexts. The Portal features a decision-tree approach for a user to choose among a suite of ES assessment tools for three different types of decision contexts: Ecological Risk Assessments (ERA), Contaminated Site Cleanups, and a more generalizable, Other Decision-Making Contexts.

An ERA process evaluates the likely environmental impacts from exposure to environmental stressors [4–6]. Site cleanups may be needed because of contamination through spills, leaks, and other impacts of hazardous materials contaminating land, ground and surface water, and indoor and outdoor air. Finally, for the Other Decision-Making Contexts pathway, ES assessments can be considered for the generic steps in structured decision-making [7]. That is, this pathway is relevant to any decision context [8].

## 2. ES Assessment Tools—Overview

The Portal was developed with and for the intended user, including the development of crosswalks between the end user’s language and descriptions of ES tool functionality to help the user select relevant and appropriate tools for their decision context. The Portal provides users with a simple process for selecting which tool is best for their specific needs. A suite of publicly available EPA tools that examine ES were reviewed for relevance to a tool selection portal designed to connect ES assessment tools to different steps in assorted environmental decision-making processes. For inclusion in the Portal, these tools needed to meet several criteria: (1) they are actively in use, completed EPA peer review, and are publicly available; and (2) had ES elements or endpoints as a key feature. A total of 11 EPA tools, frameworks, and methodologies deemed relevant for supporting environmental decision-making were included in the Portal. These include the tools described below.

### 2.1. NESCS Plus

The National Ecosystem Services Classification System (NESCS Plus (https://www.epa.gov/eco-research/national-ecosystem-services-classification-system-nescs-plus, accessed on 21 January 2024)) is a framework-based tool used to identify potential ES for a given decision context [8]. A classification system uses a standardized vocabulary [9] and the NESCS Plus uses codes to describe a taxonomy of classes and subclasses [10]. The NESCS Plus also provides a common ES language that can be useful for analyzing and communicating ES information for a given decision. The NESCS Plus has linkages to multiple EPA tools such as the Final Ecosystem Goods and Services (FEGS) Scoping Tool, the EcoService Models Library, and the EnviroAtlas) using a common language [10]. The NESCS Plus does not require extensive expertise; however, familiarity with using online, interactive databases is helpful.

The NESCS Plus can be used to answer questions like:

- What components of nature (e.g., water, fauna, flora, etc.) are being used or valued by people?
- Who are the people and economic sectors that benefit from ES?
- How do these people benefit (e.g., direct use, existence value, etc.) from ES?

The reader is pointed to a suite of existing NESCS Plus case studies (https://www.epa.gov/eco-research/national-ecosystem-services-classification-system-nescs-plus-case-studies, accessed on 21 January 2024) to learn more about potential applications including limitations or considerations of the tool [10–22].

### 2.2. FEGS Metrics Report

The FEGS Metrics Report presents a transparent approach to identify and select case-specific environmental attributes and user-specific metrics for a given decision-making context [23]. The report guides users through more than 200 metrics capturing 45 ways in that people directly benefit from ecosystems (called final ecosystem goods and services, or FEGS) in order to answer the question, “How can I identify and develop metrics for new ecosystems or beneficial uses?” No specific expertise is needed to use the report.

The FEGS Metrics Report can be used to answer questions like:

- How can I identify and develop metrics for new ecosystems or beneficial uses?
- What metrics should I use to measure final ecosystem goods and services?
- What are suggested data sources for these metrics at regional and national scales?
- Are there remotely sensed datasets or models available for these metrics at regional and national scales?

In addition to the FEGS Metrics Report (https://www.epa.gov/eco-research/final-eco-system-goods-and-services-fegs-metrics-report, accessed on 21 January 2024), the user is directed to a detailed spreadsheet of FEGS metrics for seven ecosystem types. Overall, the FEGS Metrics Report can be used in conjunction with other tools in the Portal, helping the user define very specific metrics for monitoring the condition and status of ES identified by other tools. The reader is directed to the report [23] for examples in different ecosystem types.

### 2.3. FEGS Scoping Tool

The FEGS Scoping Tool (https://www.epa.gov/eco-research/final-ecosystem-goods-and-services-fegs-scoping-tool, accessed on 21 January 2024) uses a transparent and repeatable approach for identifying and prioritizing among stakeholders, documenting the ways stakeholders benefit from the environment, and identifying components of the environment needed to realize benefits for a specific decision or decision context [24]. Familiarity with the stakeholder groups involved in a decision is the only requirement to use the tool.

The FEGS Scoping Tool can help answer questions like:

- How are stakeholder groups benefiting from the environment?
- What components of the environment are needed to realize those benefits?
- What interests do different groups have in common?

The downloadable FEGS Scoping Tool also comes with a user manual [24].

### 2.4. EnviroAtlas

The EnviroAtlas (https://www.epa.gov/enviroatlas, accessed on 21 January 2024) is an interactive web-based tool containing more than 400 environmental and social geospatial data layers that can be used for a given decision-making context [25]. The EnviroAtlas requires little expertise; familiarity with using online geospatial maps, selecting, and viewing different data layers are helpful.

The EnviroAtlas can be used to answer questions like:

- What national, community, and demographic datasets can I map at my site?
- How do the natural environment and ES vary around my site?
- How can EnviroAtlas maps and tools help me tell the story about the status of local environments, populations, and contaminated sites needing improvement?

Example applications of the EnviroAtlas can be found in [26–29].

### 2.5. EcoService Models Library

The online EcoService Models Library (https://esml.epa.gov/, accessed on 21 January 2024) (ESML) database is used to find and compare potential ecological models for quantifying ES for a given decision context [30]. The ESML requires a medium level of expertise; familiarity with ecological modeling concepts and the ability to search an online database are helpful.

The ESML can be used to answer questions like:

- What models are available for specific ES?
- What models are available for specific environment types?
- What models are available for specific Ecological Assessment Endpoints?

There are over 150 ecological models with NESCS Plus response variables such as [31–33].

### 2.6. Eco‐Health Relationship Browser

The interactive Eco-Health Relationship Browser (https://www.epa.gov/enviroatlas/enviroatlas-eco-health-relationship-browser, accessed on 21 January 2024) visually shows linkages between human health and ES through connections identified in the peer-reviewed literature [34]. This tool includes information about different ecosystem types, the services provided, and how those ES may affect people. The Eco-Health Relationship Browser does not require extensive expertise; it only requires familiarity with using an online interactive series of pathways.

The Eco-Health Relationship Browser can help answer questions like:

- What scientific evidence is available on linkages between ES and human health?
- How can green spaces such as urban forests, parks, and gardens improve human health?
- How can greening an area (for example, planting trees) affect air quality, water quality, and heat hazard mitigation?

### 2.7. EPA H_2_O

The EPA H_2_O mapping tool can be used to calculate ES for different scenarios of land use [35]. Users map ES, complete spatial queries, and generate customized reports to compare scenarios. This tool was initially developed for the Tampa Bay area but can be applied to other areas by users who have expertise using GIS.

The EPA H_2_O tool can help answer questions like:

- How do land-use scenarios at my site affect services (for example, flood protection and natural removal of pollution from the air and water)?
- How do land-use scenarios affect costs (for example, health care, wastewater treatment, and stormwater infrastructure)?
- How do land-use scenarios at my site affect flood protection?

The EPA H_2_O tool (https://www.epa.gov/water-research/ecosystem-services-scenario-assessment-using-epa-h2o, accessed on 21 January 2024) has a Basic Module and an Advanced Module. The Basic Module is intended for users with no experience with geographical information system software and can be used to generate maps and reports for areas within the Tampa Bay Region. The Advanced Module is for users who are comfortable working with spatial datasets to substitute in data from other locations and more customized scenarios.

There are several publications on the EPA H_2_O tool [36–38].

### 2.8. Practical Strategies Report

The Practical Strategies (for Integrating Final Ecosystem Goods and Services into Community Decision-Making) synthesis report (https://cfpub.epa.gov/si/si_public_record_report.cfm?dirEntryId=337461&Lab=NHEERL, accessed on 21 January 2024) describes the EPA’s Office of Research and Development’s research to connect ES benefit assessments to environmental decision making [7]. The report presents examples of place-based studies that utilized ES to inform decision making and identified best practices and gaps in those practices. No expertise is needed to use the report.

The Practical Strategies Report can be used to answer questions like:

- What are some strategies for identifying potential ES objectives?
- What are some strategies for evaluating ES trade-offs?
- What are some strategies for communicating ES risks and benefits?

### 2.9. Rapid Benefit Indicator Approach

The Rapid Benefit Indicator (RBI) approach (https://www.epa.gov/water-research/rapid-benefit-indicators-rbi-approach, accessed on 21 January 2024) allows users to estimate and quantify environmental benefits of an ecological restoration decision [39–41]. It includes a fillable checklist and a spatial analysis toolset to help users develop and summarize indicators. The RBI requires low to medium level expertise. The tool requires familiarity with or knowledge of the sites and surrounding area. The tool contains a checklist format as well as a Spatial Analysis Toolset. No expertise is needed to use the checklist, but familiarity with geographical information systems (e.g., ESRI’s desktop software, ArcMap, or ArcCatalog) aids with the Spatial Analysis Toolset.

The RBI approach can be used to answer questions like:

- How do I prioritize among restoration sites and projects?
- How can I determine who may benefit from a project?
- How can I evaluate trade-offs?

### 2.10. VELMA

The VELMA (Visualizing Ecosystem Land Management Assessments) modeling platform (https://www.epa.gov/water-research/visualizing-ecosystem-land-management-assessments-velma-model, accessed on 21 January 2024) can be used to improve the water quality of streams, rivers, and estuaries by using engineered and natural green infrastructure (GI) to reduce pollution from point and nonpoint sources. It is designed to help users assess green infrastructure options for controlling the fate and transport of water, nutrients, and contaminants across multiple spatial and temporal scales for different ecoregions and present and future climates. The VELMA platform was designed for a variety of users, including scientists, engineers, land managers and policymakers, and individual communities and stakeholder groups.

The VELMA platform requires advanced expertise; familiarity with geospatial mathematical modeling concepts is helpful. The VELMA platform can be used to answer questions like:

- How can green infrastructure impact water quality and contaminants at my site?
- How do climate scenarios affect ES at my site?
- How resilient will my green infrastructure solution be over time?

There are numerous publications and presentations about VELMA on the VELMA website [42–46].

### 2.11. CADDIS

The online CADDIS (Causal Analysis/Diagnosis Decision Information System) tool (https://www.epa.gov/caddis, accessed on 21 January 2024) for use in causal assessments of stressors on an ecosystem [47]. The tool uses a step-by-step approach to stressor identification, based on the U.S. EPA’s Stressor Identification Guidance Document, and describes additional tools and information and helpful for assessments. The CADDIS tool requires a medium level of expertise; CADDIS is designed for use by scientists and engineers familiar with ecological and environmental stressor data.

The CADDIS tool can be used to answer questions like:

- What stressors could be impacting ES at my site?
- What are the sources (land uses or entities) that directly or indirectly result in stressors at my site?
- What data analysis approaches can help me understand stressors at my site?

There are numerous publications and presentations about CADDIS on the CADDIS website [48–52].

### 2.12. Individual ES Tool Summary

Each of the 11 ES assessment tools has an overview page within the Portal that provides a high-level overview, additional references, and a hyperlink to the tool’s web page. At the top of each page is a brief description of the tool. There are three dropdown headers for each decision pathway where the user can get a snapshot of which steps in each decision pathway a given ES assessment tool could be used.

**Figure 1.**
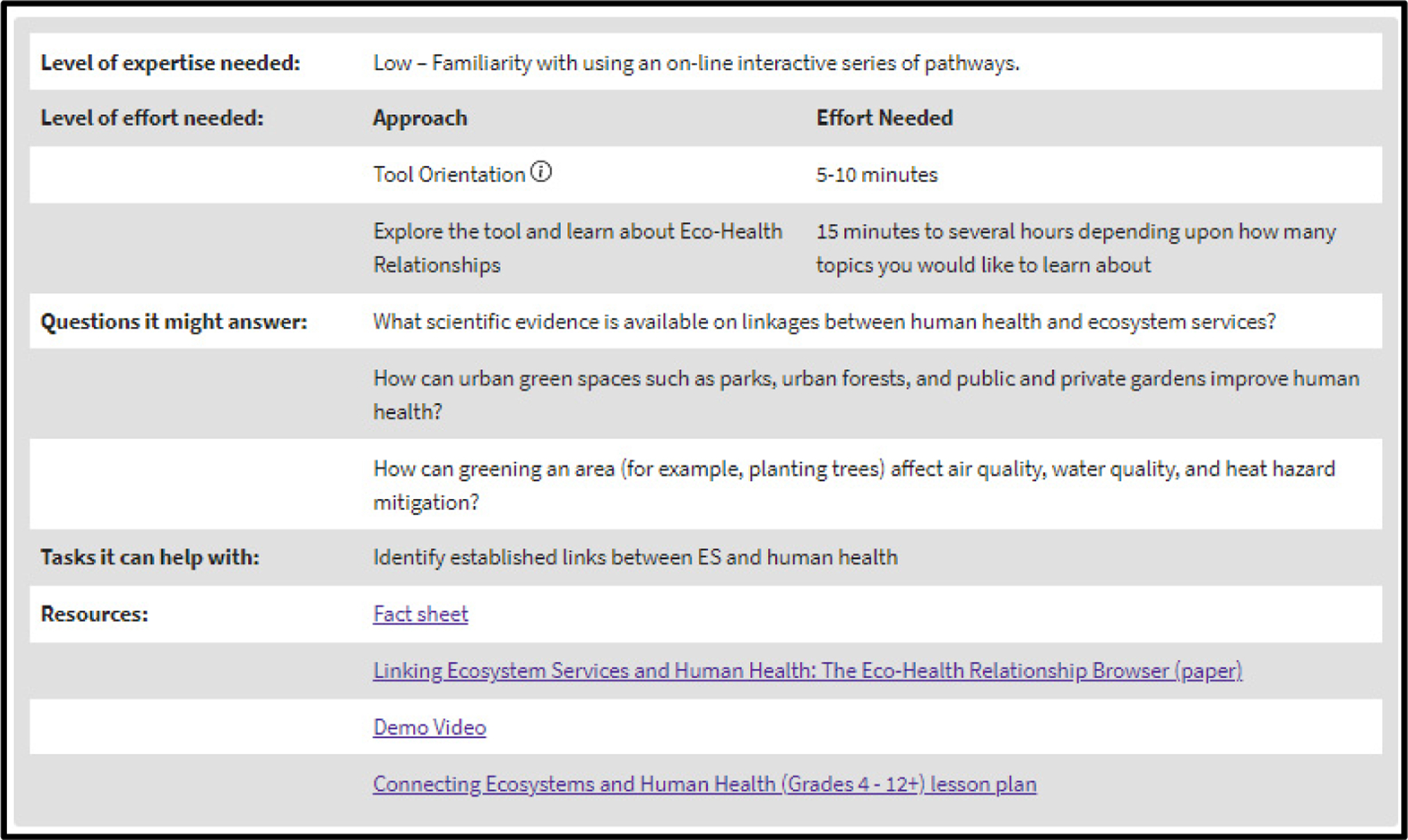
Example of the metadata summary table for the Eco-Health Relationship Browser.

A metadata summary table is at the bottom of each tool summary page (Figure 1). There, the user can learn about the level of expertise needed to use the tool, as well as information about the level of effort required. The summary table also provides some example questions that the tool might be able to answer, as well as a list of tasks the tool can help with. Finally, the summary table includes several hyperlinked resources providing more information; all point to peer-reviewed information.

## 3. The Portal

Decision support tools are designed to be used across a wide range of decision processes [53]. The Portal is designed to use the language and decision steps for three different decision processes (paths), allowing decision-makers to use familiar language to identify appropriate tools and when they might be used. Each of the three decision processes of the Portal follows a different path, has different points at which these tools would be useful, and uses different language for describing those points.

### 3.1. Using a Decision‐Tree Approach

There are a range of potential approaches for connecting ES to a given environmental decision-making context/process. In order of least to most complex, they range from:

- Using best professional judgment;
- Finding relevant examples from other efforts;
- Testing individual tool applications;
- Using a systematic, decision-tree approach to navigate among potentially relevant ES tools and frameworks relevant to that decision-making process.

The Portal represents the latter systematic, decision-tree approach. The navigation of the Portal is designed to walk the user through these sets of choices with four simple steps.

Step 1—Involves selecting one of the three paths (Ecological Risk Assessment [54–56]; Contaminated Site Cleanup, or Other Decision-Making Contexts) to start the journey. For each choice, there are several individual descriptors for the user to “Choose this path if you are: …”.

Step 2—Walks the user through answers to the question “I want help incorporating ecosystem services into…” These choices represent the process steps for that path (i.e., the five generic steps in an ERA; the five generic steps in a Contaminated Site Cleanup; and the six generic steps in structured decision making for the Other Decision-Making Contexts).

Step 3—Walks the user through identifying potential connections between a given step within their chosen decision process (pathway) and the potential utility of ES science to inform that step. This represents the language crosswalk between what an ES tool might be used for any given effort and the language of the phases of the three pathway options. The complete set of crosswalk tables for all three pathways is presented in the Supplementary Materials.

After the user navigates the first three steps, the Portal then sends the user to Step 4, a short list of potential EPA ES Assessment Tools that match their interest. There is a matching tool page for each of the 11 tools, with the overview content designed for the user as the primary audience. This matching tool page helps highlight how the tool aligns with the specific user’s need. If the user wants to explore one of the tools, a link to that tool is included.

With the use of breadcrumbs, a step-tracking progress bar, and Back/Next buttons, the Portal is designed for easy and quick navigation. Obtaining matching tool results takes only as long as the user needs to answer the questions and make choices about potentially relevant ES activities. This is helpful for a user that may want to explore more than one path for their application. For example, in brownfields, the community may not know if their site is contaminated, lightly contaminated, or not contaminated (where no cleanup is required) until they have completed their assessment. In this example, the user might explore both the Contaminated Site Cleanup and the Other Decision-Making Contexts pathways.

### 3.2. Cross Walking between ES Tools and Decision Steps

Portal developers worked with risk assessors, contaminated site cleanup practitioners, and decision scientists to develop crosswalks between individual ES tools and the specific steps for each of the three pathways. A series of co-developed workshops were held with these audiences to introduce ES concepts and tools and better understand the language used by those practitioners. This allowed the Portal developers’ work to translate between disciplines for each of the three decision pathways. For the ERA pathway, this represented an advancement of earlier work done on ES-related generic ecological assessment endpoints [57] as described in [58]. For the Contaminated Site Cleanup pathway, this represented an advancement of earlier work done on introducing ES concepts in cleanup contexts [59]. For the Other Decision-Making Contexts, this represented an advancement of earlier efforts on identifying practical strategies for introducing ES into community decision-making [7]. As an outcome of these workshops, relevant ES activities were identified for each step within each of the three decision pathways (see below for Paths 1–3).

Each of the 11 ES tools were then individually examined for their potential application/relevance for each of ES the activities identified for all three decision pathways. For a given ES tool, at least four co-authors conducted this analysis step. Investigator triangulation [60] was used to first individually interpret the same information, and then, to reach group consensus for the crosswalk results.

### 3.3. Path 1: Ecological Risk Assessments

The ERA pathway in the Portal is valuable to users who may be:

- Evaluating possible impacts of environmental stressors (e.g., disease, chemicals, or invasive species);
- Predicting the likelihood of future effects;
- Using an ERA in Remedy decisions;
- Preparing and/or reviewing ERAs.

For Step 2 of the ERA pathway, the user identifies one or more of the generic ERA steps of interest: Planning and Scoping; Problem Formulation; Analysis; Risk Characterization; and Risk Communication.

For Step 3 of the ERA pathway, the user chooses among potentially relevant ES connections by answering the question, “I want help incorporating ecosystem services into…?” and selecting from a range of options that are tied to that given ERA step (Table 1).

**Figure 2.**
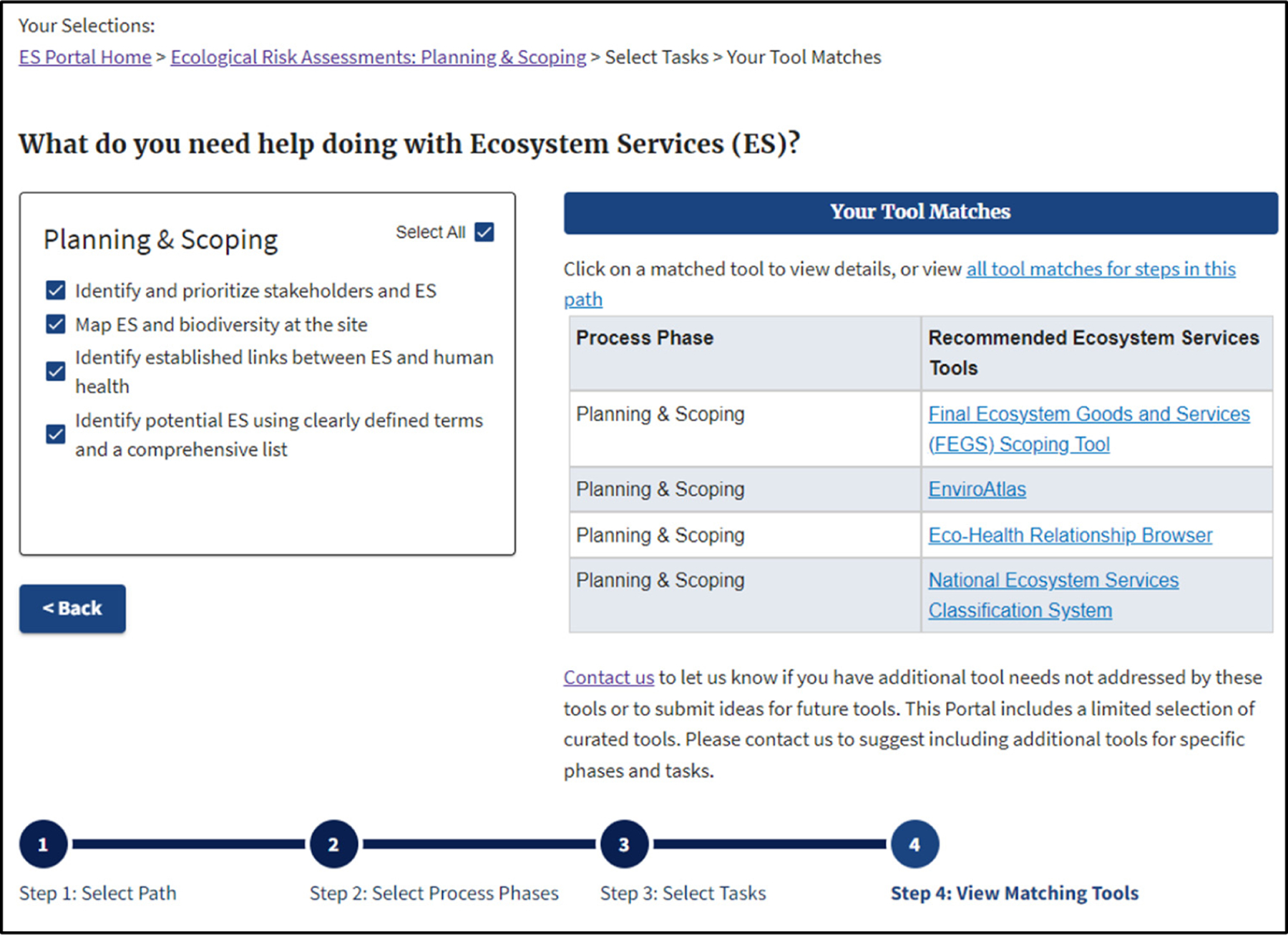
Example of matching ES Tools for all relevant ES activities in the ERA step: Planning and Scoping.

Once the user selects the radio buttons of interest, a tool matches table appears in Step 4, providing a list of ES tools relevant to those selected buttons. Figure 2 shows an example where the user chose all four potential ES connections for the ERA step in Planning and Scoping and resultant tools. Links contained within the tool matching table take the user to an overview page for a given tool, whereby the user can learn more about that tool and its potential relevance for their application.

### 3.4. Path 2: Contaminated Site Cleanups

The Contaminated Site Cleanup pathway in the Portal is valuable to users who may be:

- Doing a preliminary assessment or investigation of a contaminated site;
- Planning or engaged in cleanup or reuse of a contaminated site;
- Working with a Contaminated Site process or model.

For Step 2 of the Contaminated Site Cleanup pathway, the user identifies one or more of the generic cleanup steps of interest: Site Assessment; Site Investigation and Alternatives Evaluation; Remedy Selection; Remedy Implementation; and Post-Construction Activities. These steps were intended to be generic enough that they can be connected to specific clean-up processes (e.g., CERCLA [61], RCRA [62], Brownfields [63]).

For Step 3 of the Contaminated Site Cleanup pathway, the user chooses among potentially relevant ES connections by answering the question, “I want help incorporating ecosystem services into…?” and selecting from a range of options that are tied to that given Contaminated Site Cleanup step (Table 2).

**Figure 3.**
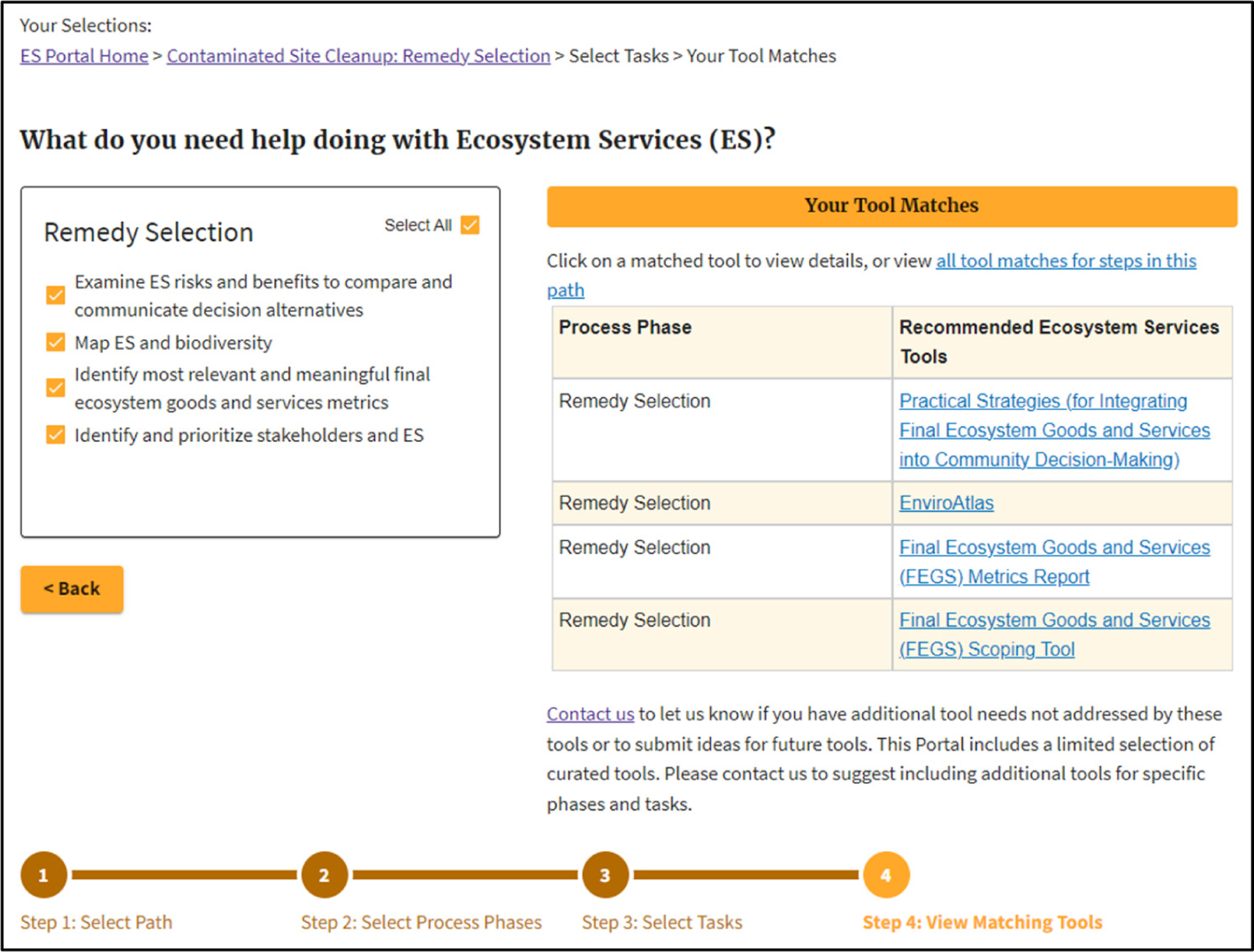
Example of matching ES Tools for all relevant ES activities in the Contaminated Site Cleanup step: Remedy Selection.

Figure 3 shows an example where the user chose all four potential ES connections for the ERA step in Remedy Selection.

### 3.5. Path 3: Other Decision‐Making Contexts

The Other Decision-Making Contexts pathway in the Portal is valuable to users who may:

- Work towards a goal that is not ERA or contaminated site cleanup (for example, natural resource management, park and recreation planning, habitat restoration, and stormwater management);
- Have a general interest in ES.

As an example, a city contemplating development of a vacant lot may want a way to evaluate proposals in a way that considers economic benefits as well as additional benefits that may result from taking a more comprehensive view of the site’s potential. In a different example, an organization may want to assess the ES impacts of past decisions to communicate their value to constituents.

For Step 2 of the Other Decision-Making Contexts pathway, the user identifies one or more of the generic Other Decision-Making Contexts steps of interest: Clarifying the Decision Context; Defining Objectives; Developing Alternatives; Estimating Consequences; Evaluating Trade-Offs and Select; and Implementing, Monitoring, and Reviewing. These generic steps match those used by [7,8].

For Step 3 of the Other Decision-Making Contexts pathway, the user chooses among potentially relevant ES connections by answering the question, “I want help incorporating ecosystem services into…?” and selecting among a range of options that are tied to that given Other Decision-Making Contexts step (Table 3).

**Figure 4.**
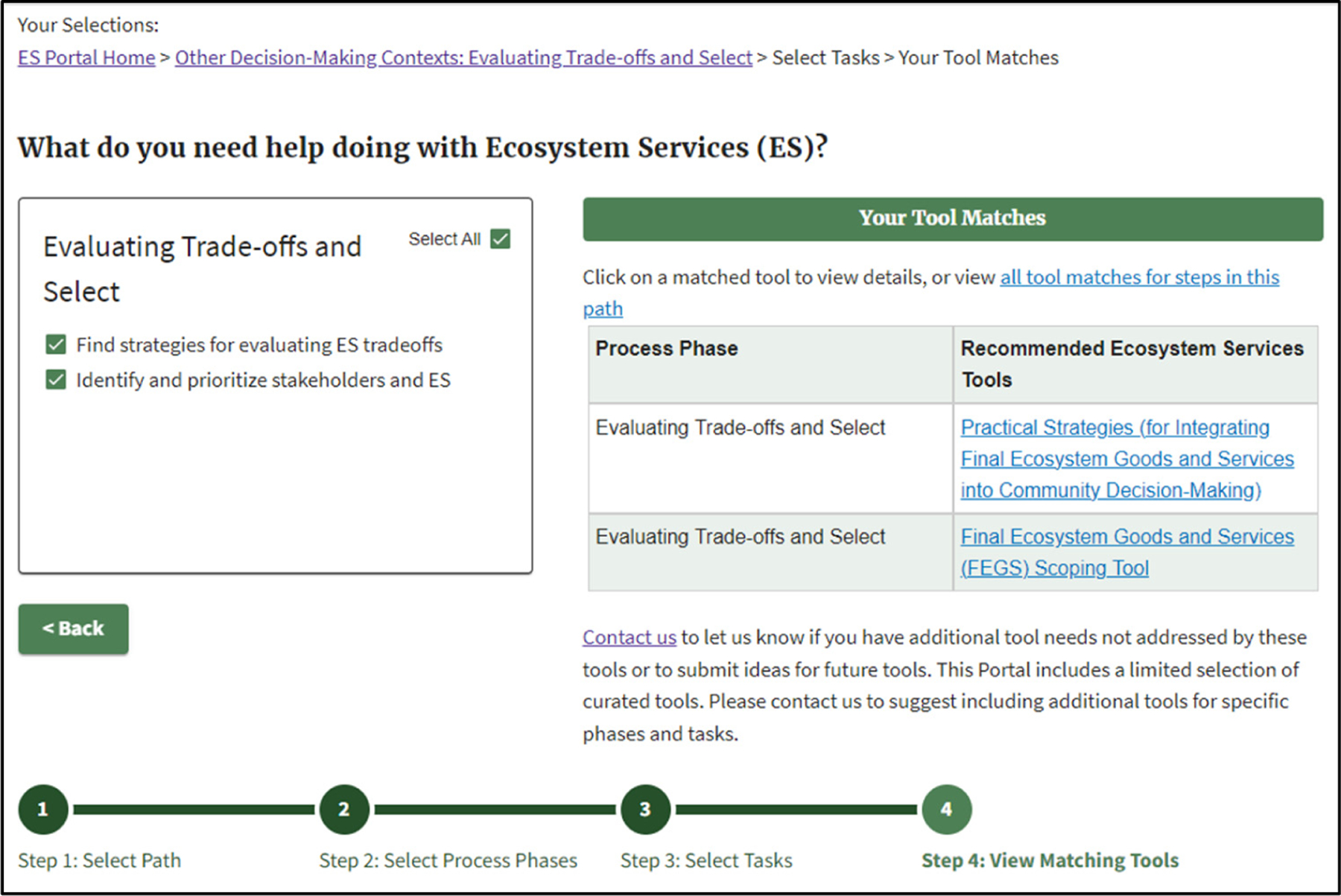
Example of matching ES Tools for all relevant ES activities in the Other Decision-Making Contexts step: Evaluating Trade-offs and Select.

Figure 4 shows an example where the user chose both potential ES connections for the Other Decision-Making Contexts step in Evaluating Trade-Offs and Select.

## 4. Example Applications

Below we present three hypothetical example applications of the Portal to demonstrate the functionality of the Portal for Ecological Risk Assessment, Contaminated Site Cleanups, and Other Decision-Making Contexts. These examples represent different types of environmental decisions, but the Portal steps were followed in a similar manner. These examples also highlight how different users may select the same tool for different needs. For example, the FEGS Scoping Tool was identified as a possible ES tool in each case study below, but for different intended purposes. In Case Study 1, it was recommended for identifying and prioritizing stakeholders in the Problem Formulation step in an ERA. In Case Study 2, it was recommended for identifying and prioritizing stakeholders in the Site Assessment and Site Investigations and Alternatives Evaluation steps in a Contaminated Site Cleanup. Finally, in Case Study 3, it was recommended for identifying and prioritizing stakeholders in the Defining Objectives step in the Other Decision-Making Contexts pathway.

The use of these tools is also likely to increase the quality of the decision process, by increasing the quantity and quality of data being considered. For example, in each of these cases, the use of the FEGS Scoping Tool requires a more structured, comprehensive, and holistic consideration of stakeholders and their relationship with the environment, economics, and societal health and well-being. Without its use, stakeholder identification is more likely to be ad hoc, overlooking less common stakeholder groups or environmental uses.

### 4.1. Case Study 1: Ecological Risk Assessment

In the first case study, decision makers wanted to determine whether to elevate the priority for cleanup of a former heavy metal mine. They were early in their process and were interested in characterizing potentially relevant ES related to the acid mine drainage footprint as part of a larger ERA. Looking at Step 1 in the Portal, the team determined that they are interested in the Ecological Risk Assessment pathway.

As the project is early in development, in Step 2, the team chose Planning and Scoping, and Problem Formulation. In Step 3, the team chose a series of potentially relevant ES activities.

**Figure 5.**
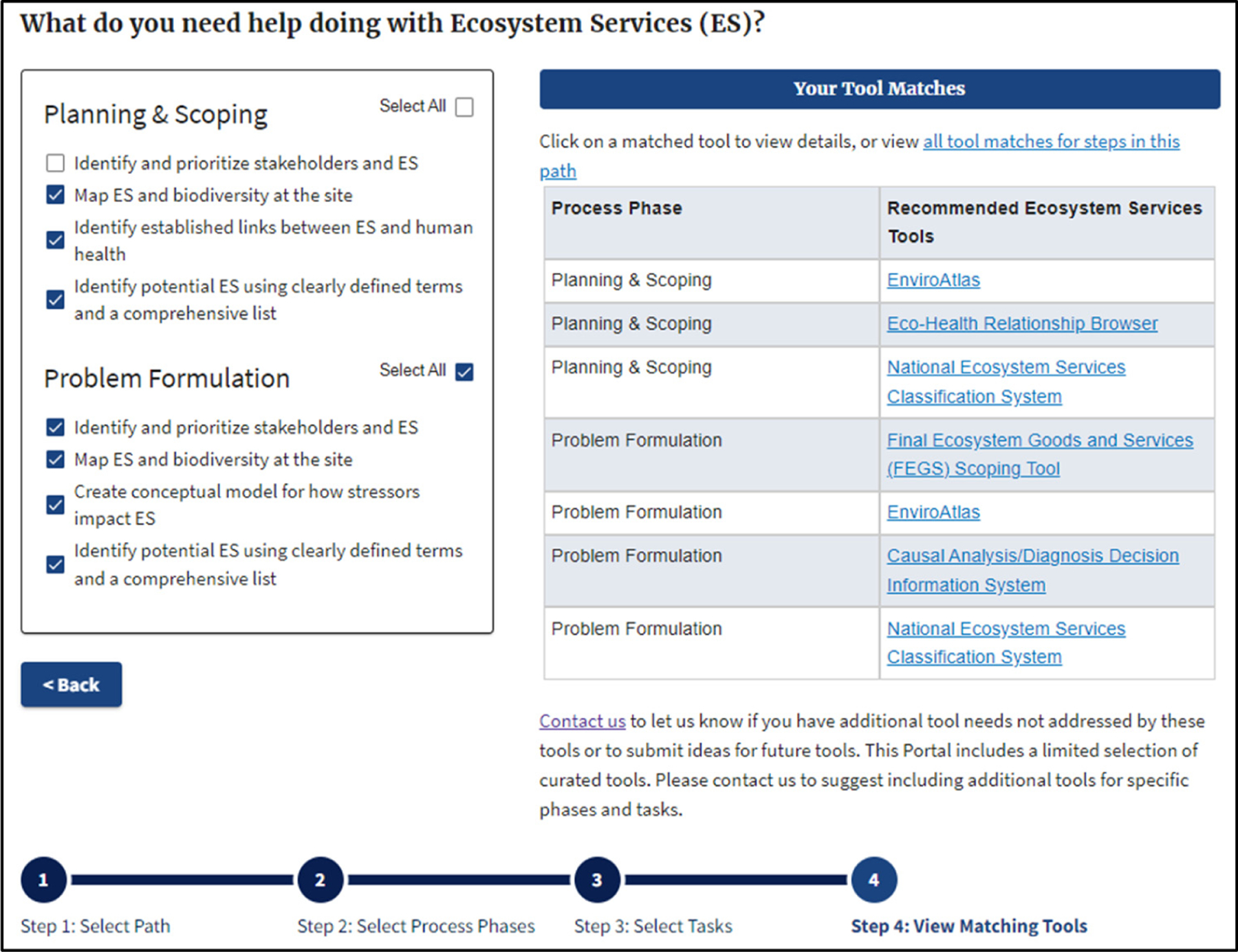
Case study results for Ecological Risk Assessment.

Step 4 yielded five potential ES tools for the team to consider (Figure 5).

The team met with several ES tool experts to determine which tools to apply and develop a strategy. These discussions included intentional efforts to focus on correct, accessible terminology as part of translating between disciplines. The team decided to apply several initially including:

- The FEGS Scoping Tool—The team’s focus would be on stakeholders, how they’re impacted by the site, and how these relationships inform a narrative for the site about whether to elevate the priority of site cleanup.
- The NESCS Plus—The team’s focus would be used to identify ES that would be reasonable to have on site after cleanup based on beneficiaries of interest, supporting the effort to develop outcome scenarios that can be used in the site’s narrative about whether to elevate the priority of cleanup.
- The EnviroAtlas—The team’s focus would be to identify aspects of the site/area as it informs a prioritization narrative. It could also be applied later as part of a deeper dive into the site, potential mitigation options, and clarification of data limitations.
- The Eco-Health Relationship Browser—The team’s focus would be to show connections between the site and human health as there appears to be more than just environmental impacts with potentially relevant connections to human health.

As for CADDIS, the team determined that they needed more information on the suite of existing environmental data at the mining site/neighboring area to examine it in the context of the CADDIS’ data and resource needs to make there were resources to apply it.

### 4.2. Case Study 2: Contaminated Site Cleanup

In the second case study, decision makers wanted to determine how to remediate the land around a former mining/smelter site to include environmental benefits (creation of a park). They were further along in the cleanup process than the first case study (at the Remedial Investigation stage) and were interested in engaging the Technical Assistance Needs Assessment group [64] to identify potential redevelopment options. Looking at Step 1 in the Portal, the team determined that they were interested in the Contaminated Site Cleanup pathway (although the Ecological Risk Assessment pathway could also be followed).

**Figure 6.**
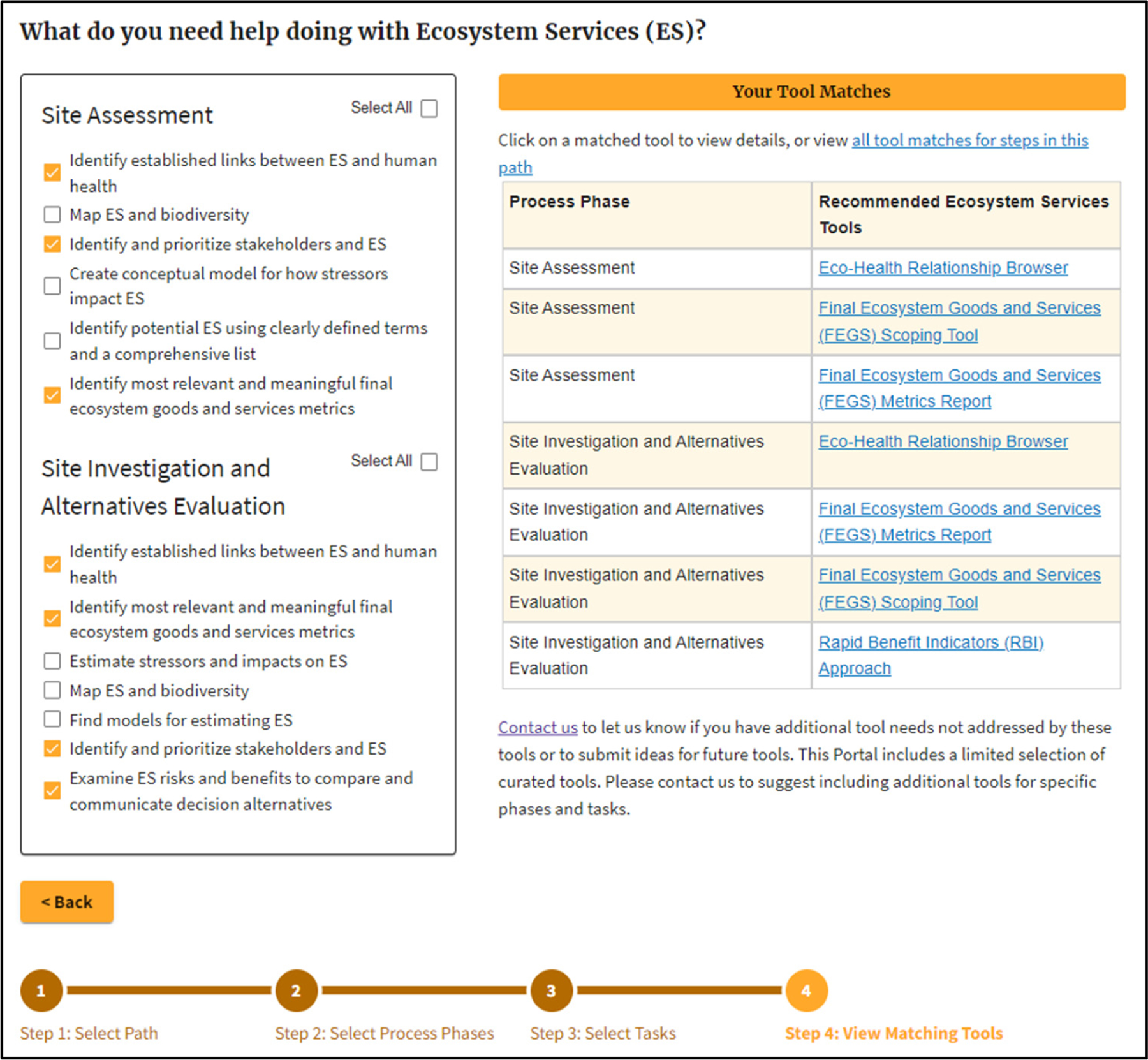
Case study results for Contaminated Site Cleanup.

As the project was further in development, in Step 2, the team chose two phases: Site Assessment and Site Investigations and Alternatives Evaluation to bracket where the site is in the cleanup process. In Step 3, the team focused their efforts translating between disciplines chose a series of potentially relevant ES activities (Figure 6, left side). Step 4 yielded four potential ES tools for the team to consider (Figure 6, right side).

Using a strategy like the first case study, the team met with several ES tool experts to determine which tools to consider based on their project’s goals (and where they were in the cleanup process). The team also decided to look for opportunities to incorporate ES tools, concepts, assessment endpoints, and models into the steps of the ERA work plan.

### 4.3. Case Study 3: Other Decision‐Making Contexts

In the third case study, decision makers wanted to further advance the consideration of reintroduction of an Endangered Species Act [65] listed species into an area that has not had that species present in a long time. Their initial assessment determined that was feasible to try a reintroduction, with the next steps involving stakeholder engagement to identify and evaluate issues around the potential reintroduction in multiple, different areas and communities. Looking at Step 1 in the Portal, the team determined that they were interested in the Other Decision-Making Contexts pathway.

**Figure 7.**
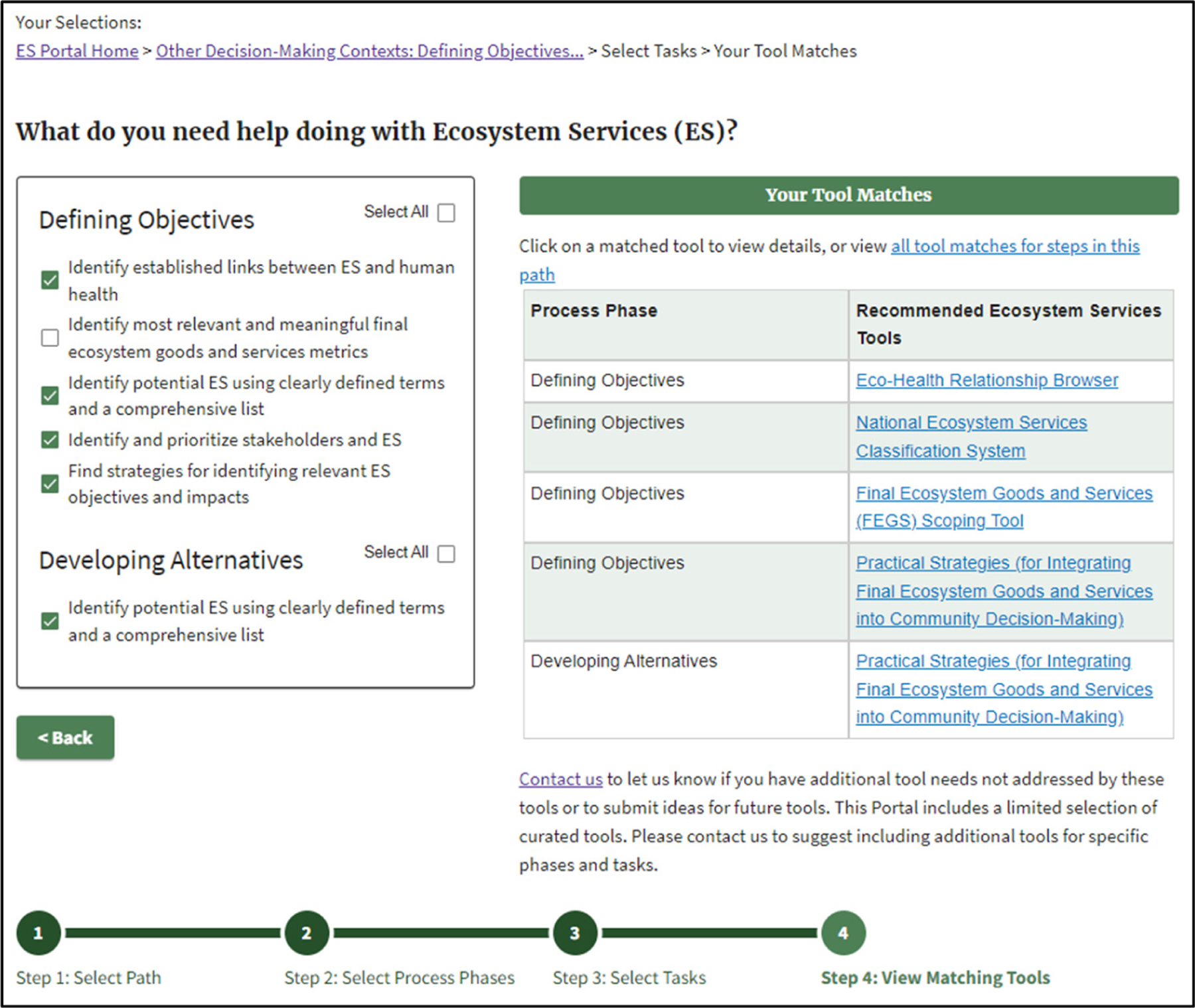
Case study results for Other Decision-Making Contexts.

As the project was early in development, in Step 2, the team chose Defining Objectives and Developing Alternatives. In Step 3, the team chose a series of potentially relevant ES activities. Step 4 yielded four potential ES tools for the team to consider (Figure 7).

The team then met with several ES tool experts to determine which tools to apply and develop a strategy. As with Case Study 1, the team included intentional efforts to translate between disciplines, determining the purpose of applying tools. The team decided to initially apply two tools:

- The FEGS Scoping Tool—The team’s focus would be on stakeholders, including ensuring they were adequately identified, and their priorities captured and communicated in a transparent manner.
- The NESCS Plus—The team’s focus would be to help identify ES affected, combining the systematic approach of NESCS Plus with information about potential ES from stakeholders and experts in the areas being examined.

## 5. Conclusions and Next Steps

When contaminated site cleanup teams have limited resources, they are often challenged to identify the resources and time required to identify tools and translate them into their own processes and needs. This Portal represents an advance in approaches to connect ES concepts to both ERAs and environmental benefits assessment of cleanup of contaminated sites. Giving contaminated site cleanup teams easy access to tools and resources to incorporate ES in their efforts enhances overall sustainable approaches for the assessment and remediation of contaminated sites.

After alpha- and beta-testing, the next steps in the development of any decision support tool involve testing the tool in a variety of applications. Even with the Spring 2023 release of the Portal, the authors have started initial application in different decision contexts, ultimately looking to learn about its: (1) effectiveness—answering the question: Did it lead the user to the right option(s)?; (2) utility—answering the question: Is it actually being used?; and (3) transferability—answering the question: How can it be applied in novel contexts? As a similar example, the EnviroAtlas is a tool that has been around a long time and has been applied in different geographic locations and contexts. And, the recently released FEGS Scoping Tool has been applied in different decision contexts and with different application purposes [11].

All the above opportunities to learn create the capacity for further tool refinement and expansion. For example, novel applications of EnviroAtlas have involved increasing the breadth and amount of available data layers. For the Portal, it presently includes only 11 ES Assessment Tools; however, there are dozens of other peer-reviewed ES assessment tools described in the literature. Future generations of the Portal may involve adding additional decision context pathways, additional ES assessment tools, and/or additional crosswalks (potentially relevant ES activities for a given step of a given decision pathway). The authors encourage readers of this article and users of the Portal to provide feedback to consider for further refinement.

## Supplementary Material

SI

## Figures and Tables

**Table 1.** Translating potentially relevant ecosystem services activities for the different steps in an Ecological Risk Assessment.

ERA Step	Relevant ES Activity
1. Planning and Scoping	Identify and prioritize stakeholders and ES
	Map ES and biodiversity at the site
	Identify established links between ES and human health
	Identify potential ES using clearly defined terms and a comprehensive list
2. Problem Formulation	Identify and prioritize stakeholders and ES
	Map ES and biodiversity at the site
	Create conceptual model for how stressors impact ES
	Identify potential ES using clearly defined terms and a comprehensive list
3. Analysis	Estimate stressors and impacts on ES
	Map ES and biodiversity
	Map alternative land-use scenarios and ES, and impacts
	Find models for estimating ES
4. Risk Characterization	Estimate stressors and impacts on ES
	Map alternative land-use scenarios and ES, and impacts
	Map pollution sources and impacts
	Examine ES risks and benefits to compare and communicate decision alternatives
	Find strategies for incorporating ES into monitoring
	Map ES and biodiversity
5. Risk Communication	Estimate stressors and impacts on ES
	Identify established links between ES and human health
	Map alternative land-use scenarios and ES, and impacts
	Identify most relevant and meaningful final ecosystem goods and services metrics
	Identify and prioritize stakeholders and ES
	Identify potential ES using clearly defined terms and a comprehensive list
	Examine ES risks and benefits to compare and communicate decision alternatives

**Table 2.** Translating potentially relevant ecosystem services activities for the different steps in a Contaminated Site Cleanup context.

Contaminated Site Cleanup	Relevant ES Activity
1. Site Assessment	Identify established links between ES and human health
	Map ES and biodiversity
	Identify and prioritize stakeholders and ES
	Create conceptual model for how stressors impact ES
	Identify potential ES using clearly defined terms and a comprehensive list
	Identify most relevant and meaningful final ecosystem goods and services metrics
2. Site Investigation and Alternatives Evaluation	Identify established links between ES and human health
	Identify most relevant and meaningful final ecosystem goods and services metrics
	Estimate stressors and impacts on ES
	Map ES and biodiversity
	Find models for estimating ES
	Identify and prioritize stakeholders and ES
	Examine ES risks and benefits to compare and communicate decision alternatives
3. Remedy Selection	Examine ES risks and benefits to compare and communicate decision alternatives
	Map ES and biodiversity
	Identify most relevant and meaningful final ecosystem goods and services metrics
	Identify and prioritize stakeholders and ES
4. Remedy Implementation	Identify most relevant and meaningful final ecosystem goods and services metrics
	Identify potential ES using clearly defined terms and a comprehensive list
	Identify and prioritize stakeholders and ES
5. Post-Construction Activities	Identify most relevant and meaningful final ecosystem goods and services metrics
	Identify potential ES using clearly defined terms and a comprehensive list
	Examine ES risks and benefits to compare and communicate decision alternatives

**Table 3.** Translating potentially relevant ecosystem services activities for the different steps in the Other Decision-Making Contexts pathway.

Other Decision-Making Contexts	Relevant ES Activity
1. Clarifying Decision Context	Identify and prioritize stakeholders and ES
	Identify potential ES using clearly defined terms and a comprehensive list
	Find strategies for identifying relevant ES objectives and impacts
2. Defining Objectives	Identify established links between ES and human health
	Identify most relevant and meaningful final ecosystem goods and services metrics
	Identify potential ES using clearly defined terms and a comprehensive list
	Identify and prioritize stakeholders and ES
	Find strategies for identifying relevant ES objectives and impacts
3. Developing Alternatives	Identify potential ES using clearly defined terms and a comprehensive list
4. Estimating Consequences	Identify established links between ES and human health
	Map people and built spaces
	Find models for estimating ES
	Create conceptual model for how stressors impact ES
	Estimate stressors and impacts on ES
	Map alternative land-use scenarios and ES, and impacts
	Examine ES risks and benefits to compare and communicate decision alternatives
5. Evaluating Trade-Offs and Select	Find strategies for evaluating ES
	Identify and prioritize stakeholders and ES
6. Implementing, Monitoring, and Reviewing	Identify most relevant and meaningful final ecosystem goods and services metrics
	Identify potential ES using clearly defined terms and a comprehensive list
	Find strategies for incorporating ES into monitoring

## Data Availability

All relevant data are within the paper and its Supplementary Materials files. Additional metadata is available at the US EP’s Science Hub: https://sciencehub.epa.gov/sciencehub.
