# Supplementary material for: The EPA Ecosystem Services Tool Selection Portal
Source: Sustainability. Author manuscript; Available in PMC 2024 Mar 20. (PMC10953757; doi:10.3390/su16051739)
Supplement: SI [file NIHMS1969135-supplement-SI.pdf]

sustainability MDPI

Supplementary Materials

The EPA Ecosystem Services Tool Selection Portal

Matthew C. Harwell [1]*, Leah M. Sharpe [2], Kaitlyn Hines [3], Cody Schumacher [3], Stephanie Kim [4], Gina Ferreira [4], and Tammy A. Newcomer-Johnson[5]

[1] Pacific Ecological Systems Division, US Environmental Protection Agency, Newport, Oregon, USA
[2] Gulf Ecosystem Measurement and Modeling Division, US Environmental Protection Agency, Gulf Breeze, Florida, USA
[3] Contractor to US Environmental Protection Agency, Cincinnati, Ohio, USA
[4] Region 2 Superfund and Emergency Management Division, US Environmental Protection Agency New York, New York, USA
[5] Watershed and Ecosystem Characterization Division, US Environmental Protection Agency, Cincinnati, Ohio, USA
* Correspondence: harwell.matthew@epa.gov

S1. Overview

In Step 4 (Matching Tools), the user can click on a pop-up link to view the full crosswalk table for each of the three decision pathways. The tables are organized by the steps for a given decision process, then by EPA ES Assessment tool and the role that tool can play (the same task label assists with the "What do you need help doing with Ecosystem Services (ES)?" question asked in Step 3.

Supplementary Table S1. Matching Tools for all Ecological Risk Assessments Steps.

Ecological Risk Assessments Step	Tool Name	Task Label
1 - Planning & Scoping	National Ecosystem Services Classification System	Identify potential ES using clearly defined terms and a comprehensive list
	Eco-Health Relationship Browser	Identify established links between ES and human health
	EnviroAtlas	Map ES and biodiversity at the site
	Final Ecosystem Goods and Services (FEGS) Scoping Tool	Identify and prioritize stakeholders and ES
2 - Problem Formulation	National Ecosystem Services Classification System	Identify potential ES using clearly defined terms and a comprehensive list
	Causal Analysis/Diagnosis Decision Information System	Create conceptual model for how stressors impact ES
	EnviroAtlas	Map ES and biodiversity at the site
	Visualizing Ecosystem Land Management Assessments (VELMA) Model	Estimate stressors and impacts on ES
	Causal Analysis/Diagnosis Decision Information System	Estimate stressors and impacts on ES
3 - Analysis	EcoService Models Library	Find models for estimating ES
	EPA H2O	Map alternative land-use scenarios and ES, and impacts
	EnviroAtlas	Map ES and biodiversity
	Visualizing Ecosystem Land Management Assessments (VELMA) Model	Estimate stressors and impacts on ES
	Causal Analysis/Diagnosis Decision Information System	Estimate stressors and impacts on ES
4 - Risk Characterization	EnviroAtlas	Map ES and biodiversity
	Rapid Benefit Indicators (RBI) Approach	Examine ES risks and benefits to compare and communicate decision alternatives

	EnviroAtlas	Map pollution sources and impacts
	EPA H2O	Map alternative land-use scenarios and ES, and impacts
	Visualizing Ecosystem Land Management Assessments (VELMA) Model	Estimate stressors and impacts on ES
	EcoService Models Library	Estimate stressors and impacts on ES
	Causal Analysis/Diagnosis Decision Information System	Estimate stressors and impacts on ES
5 - Risk Communication	Practical Strategies (for Integrating Final Ecosystem Goods and Services into Community Decision-Making)	Examine ES risks and benefits to compare and communicate decision alternatives
	Rapid Benefit Indicators (RBI) Approach	Examine ES risks and benefits to compare and communicate decision alternatives
	National Ecosystem Services Classification System	Identify potential ES using clearly defined terms and a comprehensive list
	Final Ecosystem Goods and Services (FEGS) Scoping Tool	Identify and prioritize stakeholders and ES
	Final Ecosystem Goods and Services (FEGS) Metrics Report	Identify most relevant and meaningful final ecosystem goods and services metrics
	EPA H2O	Map alternative land-use scenarios and ES, and impacts
	Eco-Health Relationship Browser	Identify established links between ES and human health
	Visualizing Ecosystem Land Management Assessments (VELMA) Model	Estimate stressors and impacts on ES
	Causal Analysis/Diagnosis Decision Information System	Estimate stressors and impacts on ES

Supplementary Table S2. Matching Tools for all Contaminated Site Cleanup Steps.

Contaminated Site Cleanup Step	Tool Name	Task Label
1 - Site Assessment	Final Ecosystem Goods and Services (FEGS) Metrics Report	Identify most relevant and meaningful final ecosystem goods and services metrics
	National Ecosystem Services Classification System	Identify potential ES using clearly defined terms and a comprehensive list
	Causal Analysis/Diagnosis Decision Information System	Create conceptual model for how stressors impact ES
	Final Ecosystem Goods and Services (FEGS) Scoping Tool	Identify and prioritize stakeholders and ES
	EnviroAtlas	Map ES and biodiversity
	Eco-Health Relationship Browser	Identify established links between ES and human health
2 - Site Investigation and Alternatives Evaluation	Rapid Benefit Indicators (RBI) Approach	Examine ES risks and benefits to compare and communicate decision alternative
	Final Ecosystem Goods and Services (FEGS) Scoping Tool	Identify and prioritize stakeholders and ES
	EcoService Models Library	Find models for estimating ES
	EnviroAtlas	Map ES and biodiversity
	Visualizing Ecosystem Land Management Assessments (VELMA) Model	Estimate stressors and impacts on ES
	Causal Analysis/Diagnosis Decision Information System	Estimate stressors and impacts on ES
	Final Ecosystem Goods and Services (FEGS) Metrics Report	Identify most relevant and meaningful final ecosystem goods and services metrics
	Eco-Health Relationship Browser	Identify established links between ES and human health
3 - Remedy Selection	Final Ecosystem Goods and Services (FEGS) Scoping Tool	Identify and prioritize stakeholders and ES
	Final Ecosystem Goods and Services (FEGS) Metrics Report	Identify most relevant and meaningful final ecosystem goods and services metrics
	EnviroAtlas	Map ES and biodiversity
	Practical Strategies (for Integrating Final Ecosystem Goods and Services into Community Decision-Making)	Examine ES risks and benefits to compare and communicate decision alternatives

4 - Remedy Implementation	Final Ecosystem Goods and Services (FEGS) Scoping Tool	Identify and prioritize stakeholders and ES
	National Ecosystem Services Classification System	Identify potential ES using clearly defined terms and a comprehensive list
	Final Ecosystem Goods and Services (FEGS) Metrics Report	Identify most relevant and meaningful final ecosystem goods and services metrics
5 - Post-Construction Activities	Practical Strategies (for Integrating Final Ecosystem Goods and Services into Community Decision-Making)	Examine ES risks and benefits to compare and communicate decision alternatives
	National Ecosystem Services Classification System	Identify potential ES using clearly defined terms and a comprehensive list
	Final Ecosystem Goods and Services (FEGS) Metrics Report	Identify most relevant and meaningful final ecosystem goods and services metrics

Supplementary Table S3. Matching Tools for all Other Decision-Making Contexts Steps.

Other Decision-Making Contexts Step	Tool Name	Task Label
1 - Clarifying Decision Context	Practical Strategies (for Integrating Final Ecosystem Goods and Services into Community Decision-Making)	Find strategies for identifying relevant ES objectives and impacts
	National Ecosystem Services Classification System	Identify potential ES using clearly defined terms and a comprehensive list
	Final Ecosystem Goods and Services (FEGS) Scoping Tool	Identify and prioritize stakeholders and ES
2 - Defining Objectives	Practical Strategies (for Integrating Final Ecosystem Goods and Services into Community Decision-Making)	Find strategies for identifying relevant ES objectives and impacts
	Final Ecosystem Goods and Services (FEGS) Scoping Tool	Identify and prioritize stakeholders and ES
	National Ecosystem Services Classification System	Identify potential ES using clearly defined terms and a comprehensive list
	Final Ecosystem Goods and Services (FEGS) Metrics Report	Identify most relevant and meaningful final ecosystem goods and services metrics
	Eco-Health Relationship Browser	Identify established links between ES and human health
3 - Developing Alternatives	Practical Strategies (for Integrating Final Ecosystem Goods and Services into Community Decision-Making)	Identify potential ES using clearly defined terms and a comprehensive list
4 - Estimating Consequences	Rapid Benefit Indicators (RBI) Approach	Examine ES risks and benefits to compare and communicate decision alternatives
	EPA H2O	Map alternative land-use scenarios and ES, and impacts
	Visualizing Ecosystem Land Management Assessments (VELMA) Model	Estimate stressors and impacts on ES
	Causal Analysis/Diagnosis Decision Information System	Create conceptual model for how stressors impact ES
	EcoService Models Library	Find models for estimating ES
	EnviroAtlas	Map people and built spaces
	Eco-Health Relationship Browser	Identify established links between ES and human health
5 - Evaluating Trade-offs and Select	Final Ecosystem Goods and Services (FEGS) Scoping Tool	Identify and prioritize stakeholders and ES
	Practical Strategies (for Integrating Final Ecosystem Goods and Services into Community Decision-Making)	Find strategies for evaluating ES tradeoffs
6 - Implementing, Monitoring, and Reviewing	Practical Strategies (for Integrating Final Ecosystem Goods and Services into Community Decision-Making)	Find strategies for incorporating ES into monitoring
	National Ecosystem Services Classification System	Identify potential ES using clearly defined terms and a comprehensive list
	Final Ecosystem Goods and Services (FEGS) Metrics eport	Identify most relevant and meaningful final ecosystem goods and services metrics
